# Decision-making and best practice when nasogastric tube feeding under restraint: multi-informant qualitative study

**DOI:** 10.1192/bjo.2022.643

**Published:** 2023-02-01

**Authors:** Sarah J. Fuller, Jacinta Tan, Dasha Nicholls

**Affiliations:** Division of Psychiatry, Imperial College London, London, UK; and East London NHS Foundation Trust, Bedford, UK; University of Oxford, Oxford, UK; Division of Psychiatry, Imperial College London, London, UK

**Keywords:** Nasogastric feeding, physical restraint, qualitative research, restrictive practices, eating disorders

## Abstract

**Background:**

Clinicians working in mental health in-patient settings may have to use nasogastric tube feeding under physical restraint to reverse the life-threatening consequences of malnutrition when this is driven by a psychiatric condition such as a restrictive eating disorder.

**Aims:**

To understand the decision-making process when nasogastric tube feeding under restraint is initiated in mental health in-patient settings.

**Method:**

People with lived experience of nasogastric tube feeding under restraint and parents/carers were recruited via the website of the UK's eating disorder charity BEAT. Eating disorder clinicians were recruited via an online post by the British Eating Disorders Society. Semi-structured interviews were administered to all participants.

**Results:**

Themes overlapped between the participant groups and were integrated in the final analysis. Two main themes were generated: first, ‘quick decisions’, with the subthemes of ‘medical risk’, ‘impact of not eating’ and ‘limited discussions’; second, ‘slow decisions’, with subthemes of ‘threats’, ‘discussions with patient’, ‘not giving up’ and ‘advanced directives’. Benefits and harms of both quick and slow decisions were identified.

**Conclusions:**

This research offers a new perspective regarding how clinical teams can make best practice decisions regarding initiating nasogastric feeding under restraint. In-patient mental health teams facilitating this clinical intervention should consider discussing it with the patient at the beginning of their admission in anticipation of the need for emergency intervention and in full collaboration with the multidisciplinary team.

Nasogastric tube (NGT) feeding against a patient's will and/or without their consent is an intervention that clinicians working in mental health in-patient units may need to implement as a life-saving treatment.^1^ The primary population in which this occurs is patients with eating disorders or other mental disorders associated with disordered eating. Any restraint, including restraint for NGT feeding, should always be a measure of last resort when best efforts to support oral nutrition fail, with subsequent deterioration in physical health. To date there is little research regarding how to implement this intervention, although there is guidance regarding dietetic aspects,^2,3^ the ethical and legal principles of NGT feeding under restraint on paediatric wards^4^ and how to modify children's nursing practice.^5^ There is also research into the patient and parent experience of this intervention^6^ and a qualitative paper exploring the experience of nursing assistants built on this work.^7^

This study is part of a larger programme of work exploring participants’ experiences of NGT feeding under restraint, which includes a cross-sectional survey across England regarding the prevalence of the intervention and the characteristics of patients requiring it in in-patient mental health settings. This is necessary because anecdotal reports identify large numbers of patients requiring long admissions when this intervention is needed but to date there is little research or guidance available for clinicians to aid best practice and consider possible prevention strategies. The overall aim of this research programme is to understand the extent of this intervention, the patient groups it most commonly occurs in and to start to identify the factors that contribute to a patient being likely to receive the intervention and factors that contribute to its discontinuation.

Shared decision-making can be defined as ‘the communication process that allows service users and service providers to collaborate when making care and treatment decisions’.^8^ Usually this is between a clinician and their patient but when working with children and young people it can also include parents.^9^ Research suggests that shared decision-making can improve both person-centred care and care quality.^10^ As NGT feeding under restraint is a restrictive intervention that occurs across the age range, understanding the decision-making process, with a focus on shared decision-making, is important when considering how to reduce the numbers of patients who require this intervention and how long the intervention is needed for. Ultimately, if clinical practice regarding shared decision-making can be improved, this may have a positive effect on patient experience, reduce associated trauma and ensure that the intervention is provided for the shortest possible time.

## Aims

We aimed to explore the decision-making process leading up to NGT feeding under restraint and to identify best practice examples.

## Method

### Design

This was a qualitative research study nested within a larger programme of work on nasogastric feeding under restraint. The methodology for the study was co-designed with the overall project steering group, which included a psychiatrist, two academics who are also psychiatrists in clinical practice, two senior mental health nurses, an eating disorders dietitian and two people with lived experience of nasogastric feeding under restraint in the context of eating disorders care, one a former patient and the other a carer. The steering group advised that individual interviews would be the most appropriate for the ‘expert by experience’ interviews, given the personal experiences the interviews would explore, with a suggested sample of five to ten participants. For the parent/carer interviews, the steering group recommended group interviews, as it was felt that the parents would get the additional benefit of peer support during the interview process. For the clinician interviews, individual interviews with 15–20 staff were considered necessary to capture the views of the range of professions within a multidisciplinary team (MDT), to include those working with children and young people as well as those working with adults, and to include both in-patient and community staff.

The research steering group met twice to discuss and then finalise the interview questions for the topic guides. These topic guides allowed the semi-structured interviews to focus on the following questions. First, there was an introductory question designed to put the participant at ease. Then the key research questions were asked; these focused on understanding the participant's experience of NGT feeding under restraint, their view on the characteristics of patients who need this intervention and suggestions to improve practice. Finally there was the opportunity for participants to talk about any area they felt was of particular relevance.

### Inclusion and exclusion criteria

Experts by experience had to have received NGT feeding under restraint in a mental health setting at some point during their treatment, be at least 1 year post discharge from mental health in-patient care and deem themselves well enough to participate in the research. They could not be involved in litigation regarding any part of their treatment. Parents/carers had to have had their loved one receive this intervention in a mental health setting. Patients (or the parents/carers of patients) who had received the intervention in paediatric or acute medical wards were excluded as the research was commissioned specifically to focus on mental health wards only. Clinicians had to work in an in-patient mental health setting where NGT feeding was carried out and to have been part of the MDT that held clinical decision-making discussions.

### Materials

Participants were interviewed using a topic guide developed with the research steering group. The topic guide was designed as a semi-structured interview with optional prompt questions, focusing on understanding the experience of NGT feeding under restraint, the impact of the intervention, the characteristics of patients who may need the intervention and how the intervention could be delivered in a way that was supportive to those who required it.

### Procedure

Recruitment was via advertising on the website of BEAT (a national eating disorders charity) and an online post by the British Eating Disorders Society (a professionals network) inviting interested individuals to contact the research team. Potential participants were sent the participant information sheet and a consent form to sign. Once signed consent forms were received, the researcher contacted the participant to arrange an interview via Microsoft Teams.

The interviews used the co-produced semi-structured topic guide and were recorded and transcribed using integrated Microsoft Teams software. At the start of each interview participants were again asked if they had any questions regarding the research and to confirm their consent verbally.

The authors assert that all procedures contributing to this work comply with the ethical standards of the relevant national and institutional committees on human experimentation and with the Helsinki Declaration of 1975, as revised in 2008. All procedures involving human participants/patients were approved by Imperial College London's Research Ethics Committee (reference number 21IC7157).

### Data analysis

This qualitative research project used thematic analysis based on the principles outlined by Braun and Clarke.^11^ Six phases were used to explore patterns and identify themes: (1) familiarisation; (2) development of the coding frame; (3) validation of the coding frame with supervisors (D.N. and J.T.) using specific examples; (4) manual coding of transcripts; (5) triangulation between different types of participant; (6) validation within the steering group.

## Results

### Participants

There were 36 participants in total, divided into three groups: people with lived experience, parents or carers, and clinicians (staff members).

#### People with lived experience

Seven females with lived experience participated and their ages, at the time of interview, ranged from 19 to 54 years. All had a primary diagnosis of anorexia nervosa and had experienced NGT feeding under physical restraint in mental hospitals. The shortest reported duration of illness was 3 years and the longest was over three decades. The number of admissions for each participant ranged from 1 to 13; the shortest admission was reported as 8 months and the longest as 5 years. Some participants they were recalling experiences that were very recent, for example 14 months ago, whereas one participant reported being fed under restraint three decades ago. Some participants were recalling experiences from both child and adolescent mental health services (CAMHS) and adult services. They recounted CAMHS admissions in general adolescent units (GAUs), psychiatric intensive care units (PICUs), low secure units (LSUs) and specialist eating disorder units (SEDUs). The adult admissions were to SEDUs. Admissions were across National Health Service (NHS) and private sector services.

#### Parents/carers

Thirteen parents participated across three group interviews. Predominantly mothers attended; however, there were two fathers and one stepmother who participated. All parents represented daughters who had experience of being NGT fed under physical restraint when aged between 12 and 27 years. One parent's daughter experienced the intervention only twice, whereas another parent reported a 7-year history of back-to-back in-patient admissions during which her daughter required the intervention multiple times for several months. The parents represented admissions in GAUs, PICUs and SEDUs in CAMHS and SEDUs in adult services.

#### Clinicians

Sixteen staff were interviewed; five were male and eleven were female. The shortest recorded clinical experience was 10 months and the longest was 17 years. Staff were from a variety of professional backgrounds: psychiatry, psychology, dietetics, occupational therapy, nursing, healthcare assistants and peer support workers. Staff represented services that spanned NHS and private units in CAMHS (GAU, SEDU, LSU, M (medium) SU, PICU) and adult services (SEDUs and LSU).

### Thematic analysis

There was considerable overlap in themes across the three samples and the results are therefore presented by theme rather than by participant group. When participants were asked how the decision to start NGT feeding under physical restraint was made, there were two clear opposing themes with six subthemes (Appendix). Each theme will be explored and illustrated with a quotation from participants.

#### Quick decisions

A number of participants, predominantly parents/carers, spoke about medical risk leading to urgent decision-making for the clinical team, where NGT feeding under restraint was considered a life-saving intervention:

‘I mean, you cannot sort of muck about when somebody is in that situation, they said she may not have 24 hours to live, you just have to get the calories into them. She was refusing what they were offering so therefore she had to have had the NG tube. When she refused that, they had to restrain her.’ (Participant 5, parent)

For some clinicians, this urgency of responding to medical risk was seen in a wider context. They felt that prevention of a potential transfer to a medical/paediatric ward because of physical deterioration was justification for a quick decision. A further reason for acting rapidly and decisively was that food refusal was affecting not just this patient, but also had wider negative impact on other patients. They explained that allowing one patient on the unit to refuse to eat had the danger of potentially leading other patients to also stop eating. For them, this impact on the patient group needed to be taken into consideration and added to the justification for quick decisions to use NGT feeding under restraint:

‘I think the psychiatrist is often coming at it from a medical point of view, not wanting to send them to paeds, and the nurses come at it from a from a ward-dynamics point of view, the impact that [someone] not eating [has on the ward] and they decide very quickly to start.’ (Participant 25, staff)

At the same time, some participants spoke about the negative effects of quick decision-making. It could lead to missed opportunities for wider MDT discussions in which perspectives from a range of disciplines inform the decision-making, with the risk that only medical risk was considered as relevant to the decision-making process. Consequently, when decisions were quick, broader risks or consequences of initiating NGT feeding under restraint were not acknowledged or understood until it was too late. The consequence could be that the patient continued needing NGT feeding under restraint for prolonged periods even after medical stabilisation:

‘I think often the MDT is [only] brought in when a mistake has been made. When NGT feeding has been implemented only based on medical risk and no wider conversations […] the patient is stuck […]. It's like the MDT gets brought in to firefight, when they really should have been involved in the decision-making process.’ (Participant 23, staff)

#### Slow decisions

Participants also described slow decisions to implement NGT tube feeding under restraint. There were two ways that these evolved. One was that the decision-making process took a long time but was coherent and thoughtful. Another was that the option of using NGT feeding under restraint was discussed repeatedly over a prolonged period without any decision to implement it, until an abrupt decision to implement it occurred.

Repeated discussions were reported by some experts by experience. They talked about being aware of discussions regarding whether NGT feeding was needed, but felt that that these had gone on for so many weeks that they were perceived as ‘empty threats’. Consequently these participants did not believe the decision to use the intervention would be made and were shocked when it was:

‘So they've been threatening it for like a long, long time, like weeks and kind of saying, like “If you're weight's not going up, then we're going to have to NG you” and then one day I left like part of my meal and like all of a sudden something changed and they just decided enough was enough.’ (Participant 16, Person with lived experience)

In contrast, one parent reported a process that reflected multiple MDT discussions over time which followed a plan of attempts to ensure that all less restrictive options had been exhausted prior to use of NGT feeding under restraint:

‘They tried everything to get her to accept it [NGT feeding] again but she refused. This went on for days and days and days with meeting after meeting after meeting. They eventually decided to restrain her and she needed that for a few weeks before she could accept the feed again.’ (Participant 6, parent)

Furthermore, some staff spoke passionately about lengthy debates within the MDT, centred on the philosophy of ‘not giving up’ on someone potentially being able to eat by themselves or accepting an NGT feed without the need for restraint. At times this was described as a unit ‘philosophy’, where there was a strong focus on collaboration with the patient regardless of how ill they were. As a result, the unit philosophy was never to rush into NGT feeding under restraint, but only arrive there after all avenues had been explored:

‘Our philosophy of care is around not giving up on anyone. In other units I've worked in when things get tricky, there's so much anxiety and they look to find a transfer option. Our philosophy is actually “This is really difficult, but we can do this” […] so we have very lengthy discussions about any level of restraint feeding.’ (Participant 30, staff)

Indeed, one clinician highlighted that at their treatment centre, discussions regarding the possible need for NGT feeding or NGT feeding under restraint started as a routine policy at admission, well before any such intervention was considered. In this process, the patient was central to the care planning in all aspects of how this could be done, including their wishes if it came to that point. Thus patient-centred collaborative care planning took place at a time when there was no conflict and no urgency:

‘We've developed our own patient-focused care planning, in which in the first week [of admission] the care coordinator sits down with the patient and runs through, in an advance directive type of way, this is how I want to be managed if it comes to NG feeding. It has graded approaches to staff support, loads of stuff about distraction techniques and if medications help etc. […] even the parents get to feed back into it and the team constantly review it.’ (Participant 31, staff)

However, one clinician talked about the negative effects of slow decision-making, the further medical deterioration and the deleterious effect this had on the patient's mental state. This led to the view in hindsight that delayed implementation of NGT feeding under restraint could mean that the patient ultimately needed NGT feeding for a longer period than if they had made a quick decision earlier on:

‘For some we took too long, usually the ones we didn't know. We would give them the benefit of the doubt and have the same discussions every week. Then we would decide “Yes, we need to do this” and they probably ended up needing the NG for longer. As a result as they […] got more ill while the decision was being made.’ (Participant 14, staff)

## Discussion

This is the first paper to qualitatively explore the decision-making process regarding the initiation of NGT feeding under restraint from multiple perspectives: those of people with lived experience, parents/carers and clinicians. The findings highlight two opposing scenarios: quick and slow decision-making. There are advantages and disadvantages to both which are explored further here, alongside what best practice might look like.

### Pros and cons of quick decision-making

When there is a high level of medical risk, there is typically clear guidance, for example when a patient with an eating disorder needs to be admitted to, or reviewed in, an acute hospital by clinicians experienced in working with eating disorders.^12^ Although such guidance typically indicates level of risk (‘red’ or high risk), it is not possible to use this guidance to identify those patients where the risk of death is imminent. Therefore, it is difficult for clinicians to identify the exact point when NGT feeding under restraint becomes a ‘life-saving treatment’. Whether a treatment is ‘life-saving’ is relevant in terms of having clear justification for imposing highly restrictive practices such as forced nutrition.

Regardless of whether there is immediate risk to life, nutrition is the relevant treatment for medical instability caused by malnutrition secondary to restrictive eating disorders. This medicalised view may appear to support quick decision-making regarding initiation of NGT feeding under restraint. However, acting on medical risk alone can be to the neglect of other aspects of risk, including psychological risk, as well as factors important in treatment and recovery, such as breakdown of trust, escalation of conflict, entrenching passive rather than active feeding, and traumatisation of the patient, family, clinical team and other patients in the unit. In slowing down decisions to impose highly restrictive/coercive measures and taking time to facilitate MDT discussion regarding the patient, clinicians may be able to see the patient in the broader context of risk versus benefit. Participants in this research felt that this was missing at times, ‘when a mistake has been made’, when NGT feeding has been implemented based only on medical risk and no wider conversations (participant 23, staff).

### Pros and cons of slow decision-making

When considering slow decision-making, participants from the staff and carer/parent groups could acknowledge that every effort to try to avoid this intervention was being taken. However, this led to the process of decision-making being perceived as meaningless ‘threats’ rather than as a considered discussion or a collaborative process with the clinical team working with the patient.

Our findings highlighted that during a slow decision-making process the patient may be deteriorating each day from both a medical and psychological perspective. One clinician highlighted the paradox that the very intervention that clinicians are trying to avoid may be needed for longer if it is delayed, as the patient becomes more physically and psychologically compromised.

### The issue of power

Cultural safety is a concept that is being increasingly used in mental healthcare.^13,14^ Cultural safety articulates often unspoken inequalities that affect patients and families, such as the power dynamic between clinicians and patients. Decisions about implementing NGT feeding under restraint is a stark example of medical authority, where mental health professionals utilise the power endowed by mental health legislation to impose nutritional intake despite lack of consent and active resistance from patients.^15^ Medical decisions of this kind, based solely on the risk of harm due to malnutrition, risk jeopardising the MDT team ethos whereby decisions are reached through consensus. Mental health social workers, for example, bring a social work perspective to decisions about using legislation to implement highly restrictive practices, and their rights-based training can be very useful in counterbalancing medical risk-based perspectives. On the other hand, medical clinicians are ultimately responsible in law, and the failure to act decisively in situations of medical risk is condemned repeatedly in coroners’ verdicts where treatment of people with eating disorders have ended in tragedy.^16,17^ Medical practitioners working in this area need support to balance the imperatives of life-saving care with the involvement of the MDT in locating that care in the wider context and ensuring that the decision-making process is owned by the whole team.

Decisions to implement NGT feeding under restraint, which by definition are made against the patient's will and involve physical holds, are likely to have both short- and long-term harms even as they seek to care for the patient and save life. The perception of repeated consideration of this intervention without action as an empty threat is concerning as there is no place for threat in compassionate mental healthcare. The perception of these discussions as threats, rather than as having their needs thought about, may reflect the extent to which patients felt excluded from the discussions.

Finally, NGT feeding under restraint also belongs in the category of highly restrictive practices under mental health (or mental capacity) legislation where many people, quite rightly, may experience moral qualms or discomfort in being part of decision-making or implementation. Just because something is legally permitted does not make it clinically appropriate, nor does it make it morally right. Every consideration of its use needs to be sensitively handled, with opportunities for staff, family members and patients to be able to think about and work through their feelings and debrief after the event or the decision. It should never become routine, nor should staff in particular become inured to the distress involved.

### Strengths and limitations

This study adds to the literature examining the decision-making process in relation to initiating NGT feeding under physical restraint. Using qualitative interviews allows for the integration of multiple perspectives and more thorough exploration than other research methods.

There are limitations to this research. First, the total number of total participants gives a good sample size, but when stratified by groups the experts by experience numbered only seven and this may not have resulted in data saturation. Second, the study gives the views of individuals in England and may not be representative of other countries. Third, although transcripts were generated by Microsoft Teams and checked against the audio recordings, there is a possibility that if the internet connection was lost briefly aspects of the interview may have been missed. Finally, all the people with lived experience reported a diagnosis of anorexia nervosa and the researchers are aware that NGT feeding under restraint can and does occur for patients with other mental health diagnoses.

### Clinical implications

This study highlights the risks and benefits of making decisions to implement NGT feeding under restraint too quickly or too slowly. In both, the timing and pace of decision-making are crucial, as clinicians need to acknowledge and understand medical risk, especially when implementing life-saving interventions in patients with eating disorders. These discussions usually occur in already difficult conditions of conflict, where patients are refusing oral intake, and there are medical consequences if refusal continues. One suggestion for good practice arising from our research is to encourage shared decision-making and involve the patient in the decision-making process from the very start of admission, so they are clear why and when a restrictive practice such as NGT feeding under restraint may be needed. Enabling the patient to discuss with clinicians their views and wishes well in advance of any conflict can inform decisions while also ensuring the patient is well informed, should the situation arise. This ‘advanced care planning’ approach involves patients, their families and the whole MDT in discussing what helps a patient to manage their required nutrition and hydration in whatever form (as food, a supplement drink or via NGT with consent) in order to avoid NGT feeding under restraint. Joint care planning on how to manage distress leading up to a restraint feed and strategies to manage distress during the intervention and to aid de-escalation after it would also help the patient and minimise the lenth of time they require the intervention. This collaborative approach, focusing on de-escalation skills, may keep the patient engaged in their treatment plan and minimise situations where conflict between the patient and the team could develop.^18^

### Future research

To our knowledge, this is the only study exploring the complexities of decision-making in real life regarding when to start NGT feeding under restraint. Future studies are needed to evaluate the clinical effectiveness of NGT feeding under restraint, the short- and long-term harms of this practice and how to minimise these harms and also explore how this morally challenging process can be needed less in future.

## Data Availability

The data that support the findings of this study are available from the corresponding author on reasonable request.

## Appendix

### Key themes and subthemes in the decision-making process

#### Theme 1 Quick decisions

Subthemes:

- medical risk
- impact of not eating
- limited multidisciplinary team (MDT) discussion

#### Theme 2 Slow decisions

Subthemes:

- perception of ‘threats’
- MDT discussion with patient
- not giving up
- advance directives
